# Pig α_1_-Acid Glycoprotein: Characterization and First Description in Any Species as a Negative Acute Phase Protein

**DOI:** 10.1371/journal.pone.0068110

**Published:** 2013-07-02

**Authors:** Peter M. H. Heegaard, Ingrid Miller, Nanna Skall Sorensen, Karen Elisabeth Soerensen, Kerstin Skovgaard

**Affiliations:** 1 Innate Immunology Group, National Veterinary Institute, Technical University of Denmark, Frederiksberg, Denmark; 2 Institute of Medical Biochemistry, Department for Biomedical Sciences, University of Veterinary Medicine Vienna, Vienna, Austria; 3 Section for Pathology, Department of Veterinary Disease Biology, University of Copenhagen, Frederiksberg, Denmark; University of Minnesota, United States of America

## Abstract

The serum protein α_1_-acid glycoprotein (AGP), also known as orosomucoid, is generally described as an archetypical positive acute phase protein. Here, porcine AGP was identified, purified and characterized from pooled pig serum. It was found to circulate as a single chain glycoprotein having an apparent molecular weight of 43 kDa by SDS-PAGE under reducing conditions, of which approximately 17 kDa were accounted for by N-bound oligosaccharides. Those data correspond well with the properties of the protein predicted from the single porcine AGP gene (ORM1, Q29014 (UniProt)), containing 5 putative glycosylation sites. A monoclonal antibody (MAb) was produced and shown to quantitatively and specifically react with all microheterogenous forms of pig AGP as analyzed by 2-D electrophoresis. This MAb was used to develop an immunoassay (ELISA) for quantification of AGP in pig serum samples. The adult serum concentrations of pig AGP were in the range of 1–3 mg/ml in a number of conventional pig breeds while it was lower in Göttingen and Ossabaw minipigs (in the 0.3 to 0.6 mg/ml range) and higher in young (2–5 days old) conventional pigs (mean: 6.6 mg/ml). Surprisingly, pig AGP was found to behave as a negative acute phase protein during a range of experimental infections and aseptic inflammation with significant decreases in serum concentration and in hepatic ORM1 expression during the acute phase response. To our knowledge this is the first description in any species of AGP being a negative acute phase protein.

## Introduction

Alpha-1-acid glycoprotein (AGP), also known as orosomucoid, is a remarkable serum protein, among the most glycosylated proteins in serum with 40–50% of its mass constituted by carbohydrate and having a very low isoelectric point due to its high content of sialic acid [1]. It has a number of microheterogenous isoforms related to variations in its carbohydrate structure and sialic acid content which are both altered in various disease states (reviewed by [2]). It also may contain a number of amino acid substitutions and in some species it is encoded by two genes (ORM1 and ORM2) both with a number of alleles and variants, as described in humans [3] and mouse [4]. In the pig one gene only has been found ([5], ORM1 (Q29014, UniProt)) having some degree of polymorphism [6]. There is extensive homology between the pig gene and the human genes, including the same numbers of putative glycosylation sites (5) and putative disulfide bonds (2). The cDNA based pig AGP sequence fragment reported by [5] is missing the two N-terminal amino acids and is 183 amino acids long; adding the missing two amino acids (Q and I, by homology to human gene) the theoretical pI and molecular weight of the pig AGP polypeptide chain is 5.83 and 21140 Da, respectively. The identification of pig AGP in classical 2-D electrophoresis, using cross-reactive anti human AGP antibodies was published recently [7]. Stone and Maurer (5) furthermore found that expression of pig AGP is developmentally regulated with high liver expression in the late stage foetus, decreasing 3–4 times in newborns and further dropping to approximately 100 times less than foetal abundance in the adult liver. This confirms other reports describing the protein as constituting up to 50% of total serum protein in newborn pigs, decreasing approximately 30 times in the adult circulation [8], [9]. This situation is the exact opposite to the one seen in humans (reviewed by [10]). Apart from the early work by Charlwood et al. [11] and the work of Lampreave and Pineiro [9] the molecular features of pig AGP have been scarcely investigated. In addition to the identification of pig AGP in 2-D electrophoresis as a microheterogeneous acidic protein [7], a ConA-binding form of pig AGP in bronchoalveolar lavage fluid (BALF) being microheterogeneous with molecular weights in the range of 40–55 kDa and a range of isoelectric points around 3–4 has been described by [12].

Although widely studied and characterized, no definitive function has been ascribed to AGP. It belongs to the lipocalin family and has the ability to bind small lipophilic/cationic molecules [13], [14]. It has immunosuppressive properties, including dampening neutrophil activation [15] and lymphocyte stimulation [16], possibly correlated to its glycosylation [17] and has also been described as having angiogenic properties [18]. The main cell type producing AGP is the hepatocyte [2], [19] but other cellular sources have also been described, notably activated neutrophils [20] and blood leukocytes [21]. It has invariably been described as a positive acute phase protein in all species studied, including human, cow, mouse, dog, cat, rabbit, rat, and chicken [1], [10], [22]. In the pig several reports propose to use pig AGP to monitor acute phase responses (e.g. [23], [24], [25], [26]).

However, Lampreave et al. [27] and Eckersall et al. [28] both described pig AGP as not changing its serum concentration during the acute phase protein response to inflammation, and this was also found by Asai et al. [29] after experimental porcine reproductive and respiratory syndrome virus infection. In addition, we recently published the surprising finding that hepatic expression of pig ORM1 was significantly decreased at 24 hours after experimental infection with the pig lung pathogen *Actinobacillus pleuropneumoniae* serotype 5b [30]. Also recently a proteomics study, looking specifically at concanavalin A-binding glycoproteins in BALF reported a local (lung), ConA-binding form of pig AGP which responded to respiratory infection with *Actinobacillus pleuropneumoniae* with either a slight decrease, a slight increase or no change in BALF concentration, depending on the pig breed (Piétrain, German Landrace and Hampshire, respectively) [12]. Yang et al. [31] also using proteomics identified a basic isoform of pig AGP to be down-regulated in serum during experimental infection of piglets with porcine respiratory and reproductive syndrome virus. However, down-regulation of AGP as such could not be demonstrated by one-dimensional Western blotting.

The acute phase protein response is a systemic serum response to infection, trauma, and inflammation, occurring 12–24 hours after tissue injury and mainly caused by changes in hepatic synthesis rates [32]. This response, although an evolutionarily old, generalized, innate defense reaction found in all metazoan species studied so-far, has some intriguing features, including the fact that acute phase proteins are found in most classes of serum proteins with the notable exception of immunoglobulin. Although the acute phase protein response in itself is highly conserved, it is made up of different serum proteins in different species [22]. Examples of this include CRP (a major human acute phase protein but not an acute phase protein in the mouse), SAA (major human acute phase protein, not an acute phase protein in the rat), and transferrin, the latter being a negative acute phase protein in man, but a positive acute phase protein in mouse [33].

Here we report a thorough molecular characterization of pig AGP purified from pooled pig serum and provide conclusive evidence both at transcriptional and protein levels that pig AGP behaves as a negative acute phase protein in the pig during specific infections as well as during inflammation-induced acute phase responses.

## Materials and Methods

### Animal Experiments and Samples

A pool of serum from Landrace pigs from conventional herds was used as the starting material for purifying pig AGP. Serum samples for quantification of pig AGP during experimentally induced acute phase responses originated from animal experiments described in [34](*Actinobacillus pleuropneumoniae* serotype 5 experimental infection), [35](aseptic inflammation), [36](*Actinobacillus pleuropneumoniae* serotype 2 and serotype 6 experimental infections), [37] (*Streptococcus suis* experimental infection) and [38] (*Staphylococcus aureus* experimental infection). Aseptic inflammation animals were Large White/Landrace/Piétrain cross-breds, pigs in the other groups were either Landrace/Yorkshire or Landrace/Yorkshire/Duroc crosses.

Furthermore, serum samples were obtained from breeding herds of Landrace, Yorkshire and Duroc breeds around 2 months of age (as described in [39]) and from SPF as well as non-SPF production herds around 20 weeks of age (described in [40]). In addition, serum samples were obtained from following types of pigs housed under standard conditions in experimental stables: Ossabaw minipigs (courtesy of Prof. Mike Sturek, Indiana University Medical School, IN, US), Göttingen minipigs (courtesy of Dr. Berit Christoffersen, Novo Nordisk A/S, Denmark), and Yorkshire/Landrace crossbreds (courtesy Dr. Jan Stagsted, Aarhus University, Denmark). Finally, serum samples were obtained from cross-breds (offspring from Danish Landrace/Yorkshire sows and Duroc or Hampshire boars) a few days after birth and from the same animals at one month of age. These pigs were either from a conventional herd or kept at the experimental stables at the National Veterinary Institute (courtesy Dr. Klara T. Lauritsen, National Veterinary Institute, Technical University of Denmark).

All animal experiments were conducted in accordance with local legislation (Animal Experimentation Act of Denmark and Ethical Committee for Animal Research at the University of Zaragoza, both in accordance with the Council of Europe Convention ETS 123). All experimental procedures involving Ossabaw minipigs were approved by the Indiana University Animal Care and Use Committee and complied fully with the Guide for the Care and Use of Laboratory Animals and the American Veterinary Medical Association Panel on Euthanasia.

### Purification of Pig AGP

Pig AGP was purified from pooled pig serum by salting out with 350 mg ammonium sulfate added per ml of serum adjusted to pH 5.5 with 1 volume of 0.2 M sodium acetate. The supernatant was collected by centrifugation, dialyzed and subjected to cation exchange chromatography (CM-Sepharose) at pH 3 at low ionic strength (0.1 M sodium acetate), yielding highly enriched pig AGP in the run-through. The more basic isoforms of pig AGP could be eluted at higher pH and higher ionic strength from the same ion exchange column. The run-through fraction was further purified by anion exchange chromatography on Q Sepharose at pH 5.5 at low ionic strength, followed by a polishing step on an S100 gel filtration column. All operations were performed with E280 monitoring and analyzing fractions by SDS-PAGE and/or Western blotting using a commercially available rabbit anti-human AGP antibody (see below).

### Preparation of Monoclonal Antibody and Development of ELISA

Purified pig AGP at approximately 1 mg/ml in PBS was used for immunizing a group of 5 mice. Briefly, the purified antigen was mixed by extensive agitation with one volume of Freund’s Incomplete Adjuvant (Difco, Detroit, MI, USA), tested for emulsion stability by floating a droplet on a water surface, and injected subcutaneously into the back of the neck of the mouse, using 100 µl (50 µg) for each injection. Three immunizations were performed with 14 day intervals. Fourteen days after the third immunization a serum sample was obtained from each mouse by tail-bleeding. The mouse sera were tested for reactivity with pig AGP in porcine serum by immunoblotting and the mouse with the best reactivity was chosen for intraperitoneal boosting with the purified antigen with no adjuvant. The mouse was killed by cervical dislocation 4 days later and the spleen immediately used for preparation of splenocytes which were then fused to myeloma cells (P3×63 Ag.8653 murine myleoma cells; ATCC,Rockville, MD, USA). Fusion and screenings were carried out essentially as described by [41] using cloning by limiting dilution in ELISA, with purified pig AGP as the positive screening antigen and serum from a boosted, immunized mouse as a positive control. The two best performing clones were selected for further cell culture, purification of monoclonal antibodies (MAbs) from the cell culture medium and tested for performance in ELISA for quantification of pig AGP. Purification of MAbs from supernatants of cell cultures grown in flasks was done by affinity chromatography on protein A-agarose (Kem-En-Tec, Glostrup, Denmark) as described previously [42]. Monoclonal antibodies were isotyped using an anti-mouse Ig subclass-specific kit (Mouse MonoAb-ID Kit (HRP), Zymed Laboratories Inc., Invitrogen). Specificity of MAbs was tested by Western blotting (see below) using unfractionated pig serum. The ELISA was optimized with respect to intra- and interassay variation and linearity under dilution, this latter point being addressed specifically as it was known that pig AGP is microheterogeneous and therefore populations having different molecular composition might react differently with the MAb. This was analyzed by two-dimensional (2-D) electrophoresis followed by blotting (see below) of pig sera with widely differing concentrations of pig AGP and by looking at parallelism of dilution curves for different pig AGP samples in the ELISA.

The final quantitative immunoassay for pig AGP was in the format of a competitive ELISA using the MAb 1.62. MAb 1.62 was coated at 1 µg/ml in 0.1 M carbonate pH 9.6 in Maxisorp microtiter plates (Nunc, Roskilde, Denmark) using 100 µl/well overnight at 4*°*C. Blocking buffer (1% bovine serum albumin (Sigma-Aldrich, Hvidovre Denmark) in washing buffer (0.05% Tween 20 in PBS)) was added at 200 µl/well and incubated for 30 minutes at standard conditions (21*°*C, on a shaking table). Then plates were washed in washing buffer (4 times) and incubated with a mix of sample at an appropriate dilution (50 µl) and biotinylated, purified pig AGP at an appropriate concentration (50 µl) i.e. 100 µl in each well. The blocking buffer was used as dilution buffer. This was incubated for 1 hour at standard conditions followed by washing as before and then horse radish peroxidase conjugated streptavidin (P397, DAKO, Glostrup, Denmark) was added at 1/2000 for 1 hour at standard conditions. After a final wash as before plates were developed with one-component peroxidase substrate solution (TMB-plus, Kem-En-Tec, Glostrup, Denmark) 100 µl/well. Color development was then stopped by adding 100 µl 0.5 M sulfuric acid to each well. For calibration, each plate contained a dilution series of a standard pig serum pool, itself calibrated against a commercially available pig AGP standard (Saikin Kagaku Institute Ltd, Sendai, Japan). All samples, including the standard were applied in duplicates. The non-optimized lower limit of quantification was 10 µg/ml. Optical densities of wells were read at 450 nm subtracting unspecific coloration at 650 nm using an automatic plate reader (Thermo Multiskan Ex spectrophotometer, Thermo Scientific, Waltham, MA, USA). Sample values were calculated from the curve fitted to the readings of the standard (using Ascent software v. 2.6, Thermo Scientific).

Pig haptoglobin was analyzed in a sandwich ELISA as described in Sorensen et al. (2006) [37]. The lower limit of quantification was 50 µg/ml as defined by the lowest concentration of the standard.

For use in the competitive immunoassay, purified pig AGP was biotinylated using biotin-N-hydroxysuccinimide (BNHS, Sigma-Aldrich Denmark, Brøndby, Denmark). Pig AGP at >2 mg/ml in 0.1 M carbonate pH 8.2 was mixed with 0.2 volumes of BNHS in N-methylpyrrolidone (2 mg/ml, fresh solution), incubated for 2 hours and dialysed against PBS overnight at 4*°*C. The working concentration of biotinylated pig AGP was determined by titrations in ELISA without competing antigen.

### Analysis of Pig AGP and Deglycosylated Pig AGP

For deglycosylation, PNGase F from QAbio (Himmelried, Switzerland) was used, following the recommendations of the manufacturer and using desialylated pig AGP as the substrate. Removal of sialic acid (N-acetylneuraminic acid) was accomplished using *Clostridium perfringens* neuraminidase (Sigma-Aldrich, N-2876) at 0.1 mg enzyme per mg of pig AGP in 0.05 M sodium acetate pH 5.2 and incubation overnight at 37*°*C.

For one-dimensional SDS-PAGE, Novex bis-tris 12%, 1 mm polyacrylamide gels were used in a Novex apparatus (Kem-En-Tec, Glostrup, Denmark) in the NuPAGE buffer system according to the manufacturer’s instructions, loading 20 µl sample in NuPAGE LDS sample buffer with reducing agent, adjusting the sample to OD (280 nm) = 0.1 (PBS) before mixing with sample buffer. Pre-electrophoresis was at 50 V, 20 minutes, followed by electrophoresis at 200 V for approx. 120 minutes. Silver-staining was performed according to the protocol of [43]. Rabbit anti-human AGP (A011), 1/500, alkaline phosphatase conjugated swine anti-rabbit IgG (D306), 1/2000, and alkaline phosphatase conjugated goat anti-mouse IgG (D486), used 1/2000, all from DAKO (Glostrup, Denmark) were employed for Western blotting performed as described previously [34]. The monoclonal antibody (MAb 1.62) was used at a concentration of 1.5 µg/ml. Blots were developed with 4-Nitro-blue-tetrazolium/5-bromo-4-chloro-3-indolyl-phosphate (NBT/BCIP) tablets (cat. no. 11 697 471 001, Roche, Hvidovre, Denmark) after the manufacturer’s instructions, terminating color development by washing the blot with several changes of MilliQ water.

Two-dimensional electrophoresis (2-D electrophoresis) was performed as a combination of isoelectric focusing in immobilized pH-gradients (IPG) in the first, and SDS-PAGE in the second dimension. For both dimensions, medium-size (140 mm×140 mm×1.5 mm) home-made gels were used, as described in [44], but in the pH range 2.5–5 for IPGs [7]. Three different conditions were applied for the IPG step: a) classical reducing-denaturing (including urea, CHAPS and DTT), b) non-reducing (classical protocol, but omitting DTT and iodoacetamide; [45]), and c) non-denaturing/non-reducing (native IPG followed by non-reducing SDS-PAGE). For simplicity, analysis conditions will further in the text be called "reduced" (a), "non-reduced" (b), and "non-denatured" (c) sample. If comparison of two serum specimens was needed on one gel, two first-dimensional IPG strips were cut to appropriate size and assembled tail to head on the same SDS-PAGE gel. 2-D study under different conditions allowed studying the protein in a completely unfolded condition (a), without intracellular disulfide bonds (b) and in a more or less native form (c), at least regarding the first dimensional step. Detection was done by silver-staining (44) or immunoblotting [46]. For the latter, following 2-D electrophoresis proteins were transferred onto nitrocellulose by semi-dry blotting. Overall protein pattern was visualized with the fluorescent protein stain ruthenium(II) tris(bathophenanthroline disulfonate)(RuBPS). After probing with specific antibody and horse radish peroxidase-conjugate, immunoreactive spots were detected on x-ray film by enhanced chemoluminescence (ECL, GE Healthcare Life Sciences, Munich, Germany).

### Gene Expression Analysis

For gene expression analyses (*Actinobacillus pleuropneumoniae* serotype 2 and 6, and the *Staphylococcus aureus* experiments), RNA extraction, primer design, cDNA synthesis, preamplification, and qPCR conditions have been described previously in (30, 38). Briefly, RNAlater (Qiagen) stabilized liver tissue was homogenized and total RNA was extracted using RNeasy (Qiagen, Denmark) or Trizol (Invitrogen, Denmark). Purity and quantity of extracted total RNA was assessed by UV absorption on a Nanodrop ND-1000 spectrophotometer (Saveen and Werner AB, Limhamn, Sweden). RNA integrity number was measured on an Agilent 2100 Bioanalyzer (Agilent Technologies, Nærum, Denmark) using the RNA 6000 Nano Kit. Extracted RNA was converted into cDNA by reverse transcription of 500 ng total RNA using the QuantiTECT Reverse Transcription kit (Qiagen). Pre amplification was done using TaqMan PreAmp Master Mix (Applied Biosystems, Foster City, CA). Pre amplified cDNA was diluted at least 1∶4 in low EDTA TE-buffer (VWR – Bie & Berntsen) before qPCR. QPCR was performed in 48.48 Dynamic Array Integrated Fluidic Circuits (Fluidigm Corporation, CA, USA) combining 48 preamplified samples with 48 primer sets for 2304 individual and simultaneous qPCR reactions. The following cycle conditions were used: 2 min at 50°C, 10 min at 95°C, followed by 35 cycles with denaturation for 15 s at 95°C and annealing/elongation for 1 min at 60°C. Melting curves were generated after each run to confirm a single PCR product (from 60°C to 95°C, increasing 1°C/3 s.). Data were acquired using the Fluidigm Real-Time PCR Analysis software 3.0.2 (Fluidigm Corporation). Pre-processing and relative quantification of expression data were done as previously described [47]. Briefly, several stably expressed reference genes were used for normalization. These genes were validated using both GeNorm [48] and NormFinder [49] and included hypoxanthine phosphoribosyl-transferase 1 (HPRT1), TATA box binding protein (TBP), tryptophane 5-monooxygenase activation protein, zeta polypeptide (YWHAE), and peptidylprolyl isomerase A (PPIA) (for the *Staphylococcus aureus* experiment) and HPRT1 and glyceraldehyde-3-phosphate dehydrogenase (GAPDH) for the *Actinobacillus pleuropneumoniae* experiment.

Results of relative expression levels were compared and tested for statistical significance using Mann Whitney’s non-parametric two tailed test for the *Actinobacillus pleuropenumoniae* experiment. For the *Staphylococcus aureus* experiment the control group was too small (n = 2) to allow statistically based comparisons to be made.

## Results

### Identification, Purification and Characterization of Pig AGP

**Figure 1:**
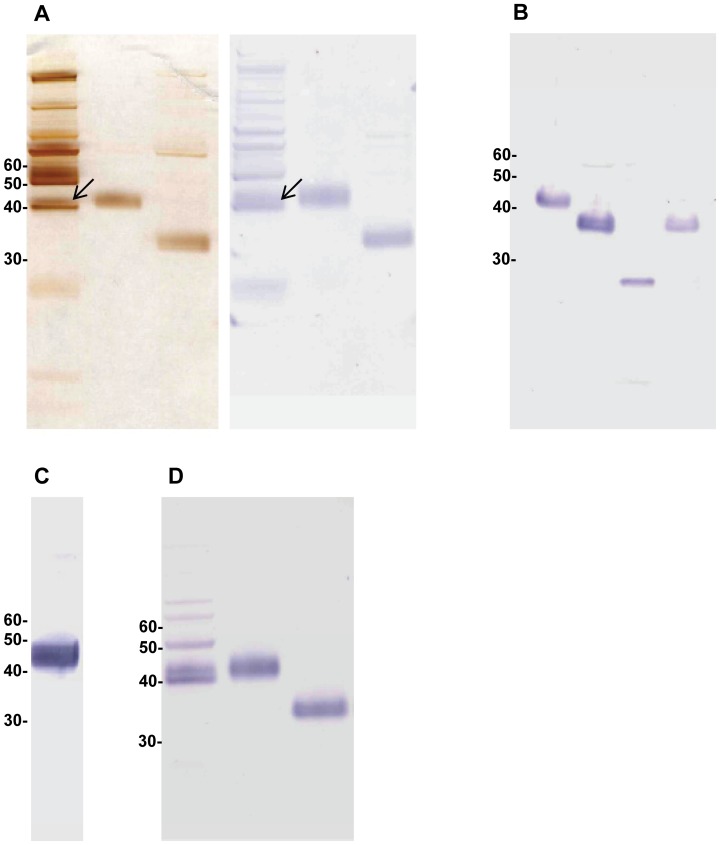
Characterization of pig AGP by SDS PAGE and Western blotting. A: Left panel: Silver-stained SDS PAGE, from the left: Salted-out pooled pig serum supernatant; purified pig AGP; purified pig AGP after sialidase treatment. Right panel: Western blot with the same samples probed with rabbit anti human AGP (DAKO). Arrow: Position of pig AGP in salted-out serum supernatant. B: Western blot probed with anti human AGP, from the left: Purified pig AGP; purified AGP after sialidase treatment; purified AGP after sialidase and PNGase F treatment; buffer control for PNGase F treatment. C: Western blot of pooled pig serum probed with antiserum (1/500) from mouse immunized with purified pig AGP (see text)(representative example). D: Western blot probed with MAb 1.62, from the left: Pooled pig serum; purified pig AGP; purified pig AGP after sialidase treatment.

In silver-stained SDS-PAGE and Western blotting purified pig AGP appeared under reducing conditions as a single broad band with a mean apparent molecular weight around 43 kDa, as indicated by its reactivity with rabbit anti-human AGP (Fig. 1A, left panel). With this antibody, unspecific reactions with a number of unrelated serum proteins were seen in salted-out serum, however the identity of this band was confirmed by the identical position and appearance of purified pig AGP in reducing SDS-PAGE and in Western blotting (Fig. 1A, lane 2, both panels). Sialidase treatment of purified pig AGP shifted its apparent molecular weight to 36 kDa, appearing as a single broad band that was still fully reactive with the rabbit anti-human AGP antibody in Western blotting (Fig. 1A, lane 3, both panels). Subsequent PNGase treatment left an immunoreactive narrow band at an apparent molecular weight of 26 kDa (Fig. 1B, lane 3). Finally, a high-titered, highly specific mouse antiserum was obtained after immunization with purified pig AGP (Fig. 1C), and the monoclonal mouse anti-pig AGP antibody subsequently developed from this mouse was shown to react with unpurified, purified as well as sialidase treated purified pigAGP in Western blotting (Fig. 1D). In both Western blots with serum (Fig. 1A and Fig. 1D) staining of higher molecular weight bands is seen, probably representing unspecific staining by the secondary antibody.

**Figure 2:**
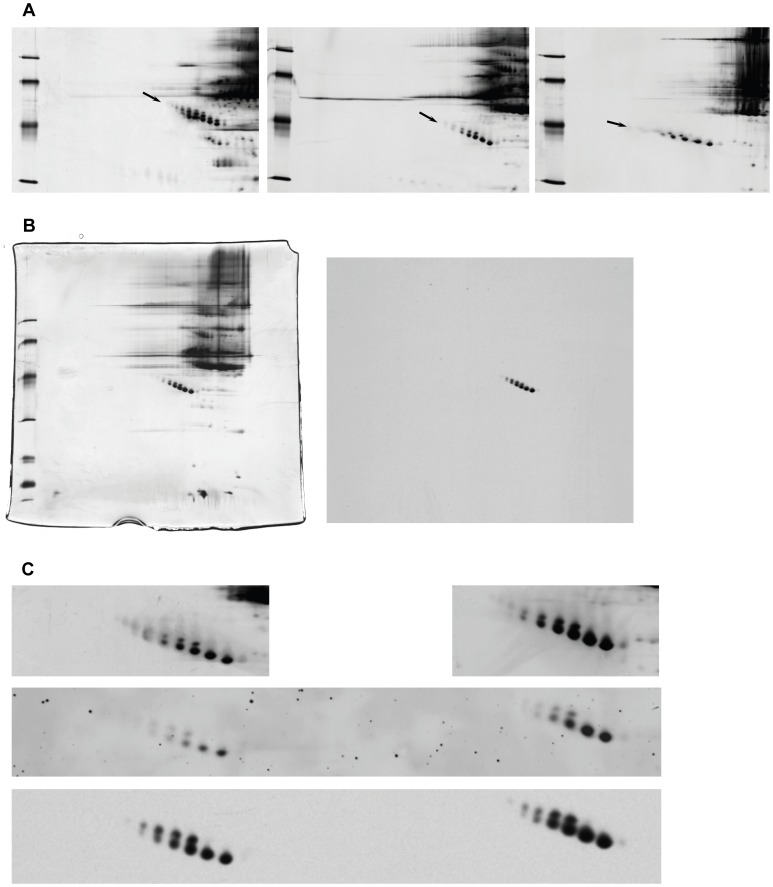
Characterization of pig AGP by 2D electrophoresis and 2D blotting. A: Pig AGP 2-D electrophoresis, influence of sample preparation conditions, from left to right: reduced sample, non-reduced sample, non-denatured sample. Close-up from gels with pH gradient 2.5–5, individual pig serum sample (B16/221). Mw markers: 30,43,67,94 kDa (from bottom). Arrow: Pig AGP isoforms. B: The reaction of MAb 1.62 with non-purified pig PAGP isoforms by 2-D electrophoresis. Individual pig serum sample (Aus), non-reducing, complete gel/blot with IPG pH 2.5–5 in the first dimension, left: silver-stained, right: blot probed with MAb 1.62. Mw markers: 14, 20, 30, 43, 67, 94 kDa (from bottom). Arrow: Pig AGP isoforms C: Close-up of 2-D electrophoresis of two individual pig sera (827 µg/ml (left), 1692 µg/ml (right)), silver-stained (top), blotted and stained by RuBPS (general protein stain)(middle), and the same blot subsequently probed with MAb 1.62 (bottom). Non-reducing, pH gradient 2.5–5. Arrow: Pig AGP isoforms.

By 2-D electrophoresis under reducing conditions pig AGP was identified as one of the most acidic pig serum proteins having several isoforms spanning the molecular weight range 44–55 kDa and the pI range 3.6 to 4.3. The high molecular weight isoforms had the low pI and low molecular weight isoforms had higher pIs, giving the pig AGP isoform population a characteristic downwards “slope” going from low pI to high pI (Fig. 2A, arrow). When run under non-reducing conditions the pig AGP population showed two molecular weight subpopulations, one slightly little less abundant population with a molecular weight 2–4 kDa higher than the other and lacking the most basic pI forms (Fig. 2A, middle panel). The pI differences between the different isoforms were similar for both populations. The MAb 1.62 reacted specifically and indiscriminately with all pig AGP isoforms (Fig. 2B and 2C) and reliably reproduced the pattern and intensity of spots in two serum samples having a two-fold difference in pig AGP concentration as seen with general silver-staining of the gel or RuBPS staining after blotting (Fig. 2C). Reduced porcine AGP had a higher apparent molecular weight than non-reduced by 2-D electrophoresis (Fig. 2A, left and middle panel, respectively) suggesting the presence of one or more intra-molecular disulfide bonds in the molecule. Omission of denaturing compounds in IPG prior to non-reducing SDS-PAGE changed the AGP pattern to less distinctly separated isoforms, a lower molecular weight slope, and markedly lower pI (Fig. 2A, right panel).

Purified pig AGP comprised the full population of isoforms; upon desialylation the slope was completely lost, and the whole population moved to a lower molecular weight (around 40 kDa) and a slightly higher pI (in the 4.3–4.5 range)(not shown), however a division into two populations was still apparent. This pattern was retained after PNGase treatment, however at a molecular weight from 26–28 kDa (not shown).

### Antibody Reactivity

After immunization with unmodified, purified pig AGP, four out of five mice yielded high titers of pig AGP specific antibodies (an example of mouse antiserum reactivity is shown in Fig. 1C). A desialylated version of the same pig AGP preparation was also used for immunization in five additional mice, however yielded antisera of lower titer towards intact porcine AGP and no cloning was attempted from these mice. Subsequent establishment of hybridoma cell lines using spleen cells from the most highly reacting mouse yielded several hybridoma clones producing monoclonal antibodies (MAbs) reacting specifically with pig AGP. One clone (MAb 1.62) of the IgG_1_ subtype with type κ light chains was used to probe Western blots and showed similar reactivity with pig AGP in unfractionated pig serum, purified pig AGP and desialylated purified AGP (Fig. 1D), closely resembling the reactivity of the polyclonal rabbit anti human AGP antibody (Fig. 1A and B) although with higher specificity. Non-reduced samples were detected with higher sensitivity than reduced samples (not shown), which was a clear advantage for the planned application of the antibody in ELISA. Therefore, also 2-D electrophoresis for Western blot testing of this antibody was performed under these conditions. Thus separated pig serum demonstrated the specificity (Fig. 2B) and the reactivity of MAb 1.62 with all pig AGP isoforms visible by silver-staining, reliably reflecting the concentration of pig AGP in two different pig sera (Fig. 2C).

### ELISA

**Figure 3:**
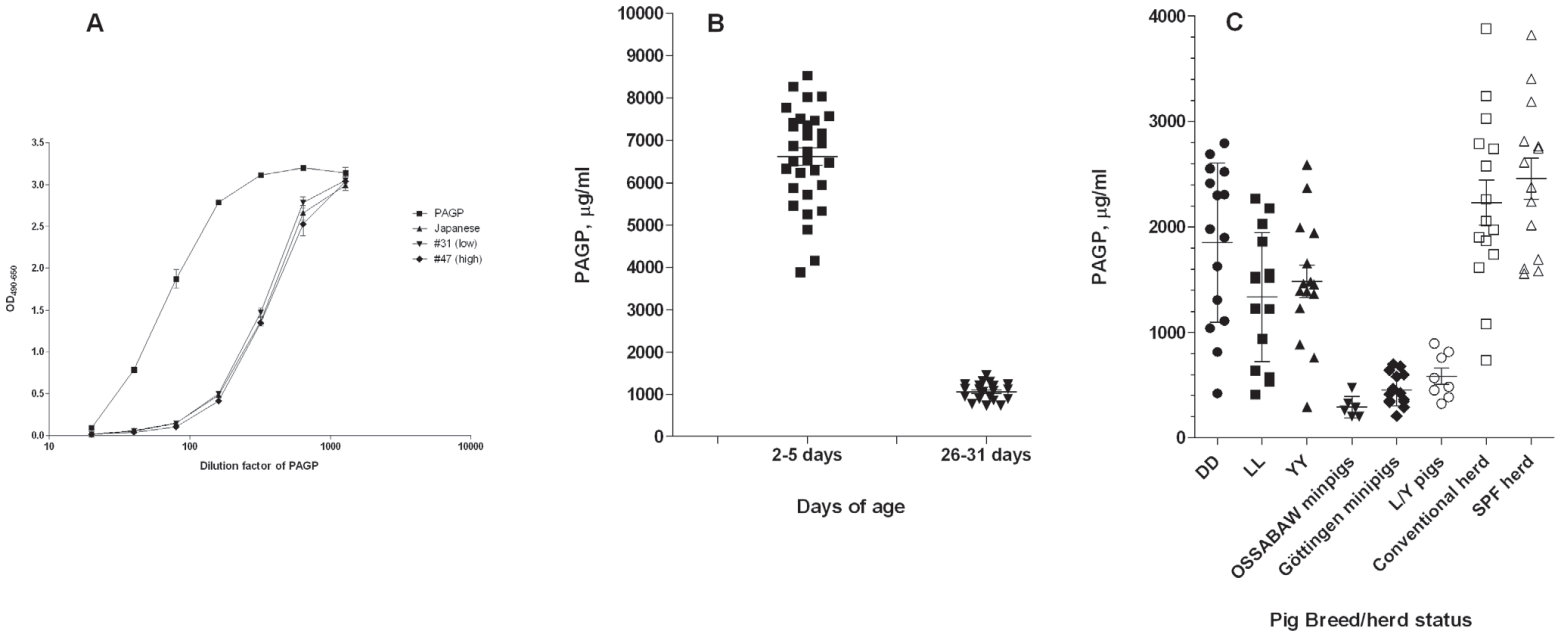
ELISA quantification of pigAGP. A: Titration of purified pig AGP, pig AGP standard (Saikin Kagaku Institute Ltd.), and two individual pig sera in competitive MAb 1.62 based ELISA. B: Serum concentrations of pig AGP in newborn piglets and in 1-month old piglets (Landrace, Duroc, Yorkshire crossbreds, N = 31). Bars indicate mean and SEM. C: Serum concentrations of pig AGP in different pig breeds and rearing conditions (individual samples), mean and SEM shown. DD: Duroc (2 months, herd) LL: Landrace (2 months, herd) YY: Yorkshire (2 months, herd) Ossabaw minipigs, Experimental stables (14–16 months of age) Göttingen minipigs, Experimental stables (41–47 months of age) L/Y: Landrace/Yorkshire crossbreds (experimental stables, 8–9 months of age) Conventional herd (5 months, D/L/Y cross bred production pigs) SPF herd (5 months, D/L/Y cross bred production pigs).

It was first attempted to develop a classical sandwich ELISA for quantification of pig AGP, however we did not succeed in identifying mutually non-excluding MAb pairs. Instead, a competitive format was validated, in which biotinylated, purified pig AGP was co-incubated with sample, being blocked in its binding to the catching antibody by any pig AGP present in the sample. MAb 1.62 was used as the catching antibody, as described in detail above (Materials and Methods). The assay proved robust and precise and showed parallel titration curves with all samples tested, including the standard (Saikin Kagaku Institute Ltd.), allowing for accurate quantification over a large dilution range (Fig. 3A). As described above, to test whether there was any bias in the reaction of MAb 1.62 with the isoforms of pig AGP, a 2-D electrophoretic analysis was run with two individual pig sera side by side, one having a high (1692 µg/ml) and another one a lower concentration of AGP (827 µg/ml) as measured by the ELISA. As can be seen in Fig. 2C, there was a good agreement between the relative silver staining intensities of pig AGP in the two samples on one hand and the relative staining intensities seen after blotting with general protein staining (RuBPS) as well as with MAb 1.62 immunostaining on the other hand, all of these reflecting the concentration differences as determined by the ELISA. In the blot it was also evident that MAb 1.62 reacted similarly with all visible molecular species of pig AGP, reproducing to large extent the relative intensities seen by silver-staining (Fig. 2C). This was also the case when – instead of non-reducing conditions – non-reducing/non-denaturing conditions were applied in 2-D electrophoresis (not shown).

### Serum Concentrations of Pig AGP

The non-biased reaction of MAb 1.62 with all pig AGP molecular species at different concentrations was reflected in the parallelism of dilution curves seen with two individual pig sera, a commercial standard preparation (Saikin Kagaku Institute Ltd.) and pig AGP purified in our laboratory (Fig. 3A).

Serum concentrations in young (2–5 days old) cross-bred piglets from a conventional herd and from experimental stables were all decreased by a factor of around 6 at day 26–31, with the mean concentration in young animals (N = 31) being 6.6 mg/ml and in the same 1 month old animals being 1.1 mg/ml (Fig. 3B).

In production pigs (Duroc (DD), Landrace (LL) and Yorkshire (YY) purebreds) from conventional breeding herds, pig AGP serum concentrations showed quite a large variation with means of 1.8, 1.3 and 1.5 mg/ml, respectively, in 2 months old LL, DD and YY pigs (Fig. 3C). Similarly, in conventional production herds and specific pathogen free (SPF) herds a rather large variation was seen and the means were 2.2 (non-SPF) and 2.5 mg/ml (SPF), i.e. a little lower, although not significant, in conventional herds compared to SPF herds. In two minipig breeds (Ossabaw and Göttingen, respectively) both mean and variance were much lower (Ossabaw: 0.3 mg/ml, Göttingen: 0.5 mg/ml) and this was also the case for Yorkshire/Landrace crossbreds kept under experimental conditions (mean 0.6 mg/ml) (Fig. 3C).

### Pig AGP Serum Concentrations During Infection and Inflammation

**Figure 4:**
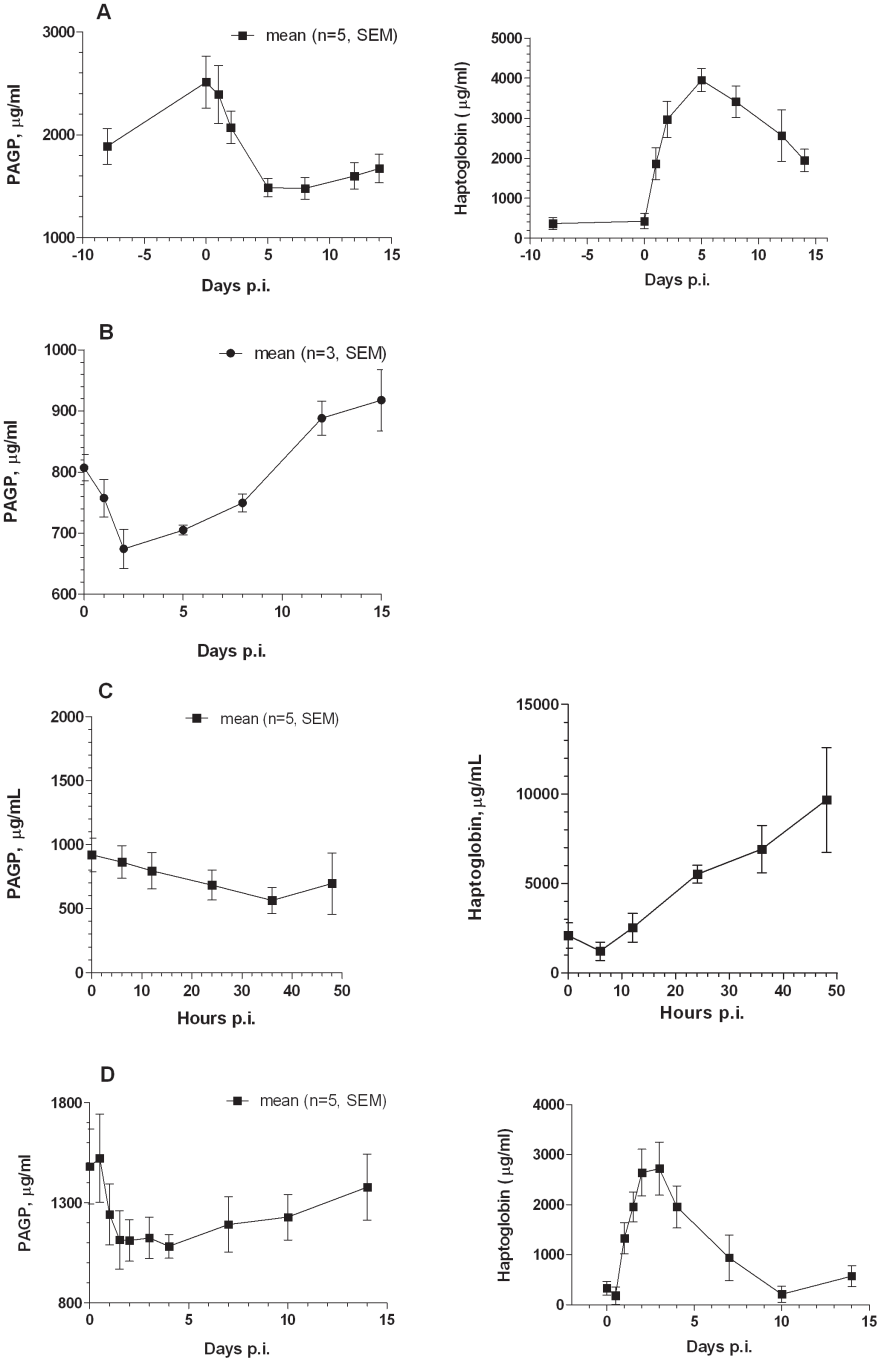
Serum concentrations of pigAGP during the acute phase response. Serum concentration of pig AGP (left) and pig haptoglobin (right) at different days post infection after experimental *Streptococcus suis* (A), *Actinobacillus pleuropneumoniae* (haptoglobin data not included) (B), and *Staphylococcus aureus* (C) infection and after aseptic inflammation (D). Note: In the *Staphylococcus aureus* experiment, only two infected pigs were sampled at 48 hours.

In experimental *Streptococcus suis* (Gram positive bacterium) infection as well as in experimental aseptic inflammation induced by subcutaneous turpentine injection (Fig. 4A and 4B, respectively) the haptoglobin acute phase response (approximately 20 fold increase) was mirrored by a substantial decrease in pig AGP serum concentrations, peaking at 5 days (*S. suis*) and 2 days (inflammation) after challenge. In the *S. suis* experiment pig AGP dropped from a day 0 concentration of around 2.5 mg/ml to appr. 1.4 mg/ml at day 5 and inflammation caused a drop from 1.5 mg/ml to 1.1 mg/ml at day 2 in these pigs (Fig. 4A and 4B). Experimental infection with the respiratory Gram negative pathogen *Actinobacillus pleuropneumoniae* also caused a decrease in pig AGP serum concentrations, concomitantly with an increase in haptoglobin (Fig. 4C and (33)), dropping from around 0.8 mg/ml to 0.6 mg/ml at day 2 p.i. With the Gram positive pathogen *Staphylococcus aureus* (Fig. 4D) a 3–5 fold increase in haptoglobin at 30–48 hours after challenge was accompanied by a small decrease in pig AGP, going from a mean in 5 animals around 1.1 mg/ml to 0.7 mg/ml, however with a big animal to animal variation.

### Pig mRNA AGP Expression in Liver

**Figure 5:**
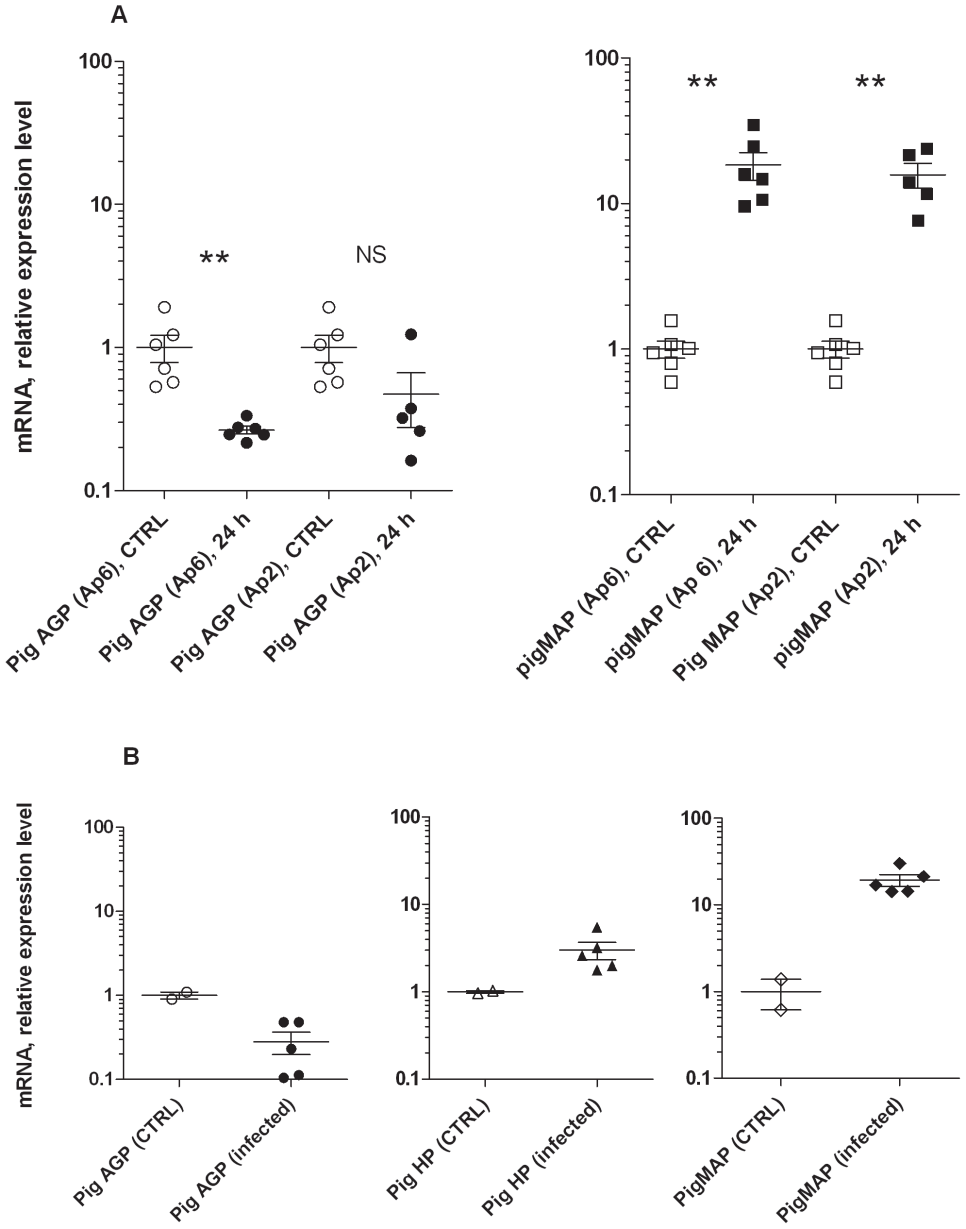
Hepatic expression of pig AGP gene during acute infection. A: Relative expression levels of pig AGP (left) and pigMAP (right) (mean of controls (CTRL, N = 6) set to 1) at 24 hours after experimental infection with *Actinobacillus pleuropneumoniae* serotype 6 (Ap6) and serotype 2 (Ap2), respectively, as indicated. Values for all individual animals are shown. Error bars depict SEM. Analysis was done on liver tissue samples by qPCR (see text). P<0.01: **, not significant: NS. B: Relative expression levels (mean of controls (CTRL, N = 2) set to 1) in *Staphylococcus aureus* liver samples 30 (N = 3), 36 (N = 2) and 48 (N = 2) hours after i.v. infection with the bacterium as determined by qPCR, pig AGP (left), pig MAP (middle) and haptoglobin (right). Controls received sterile isotonic saline and were euthanized at 48 hours. Values for individual animals are shown. Error bars depict SEM.

Concurrently with a 10–20 times increase in the relative expression of the pigMAP gene (*ITIH4*) a down-regulation of the pig AGP gene (*ORM1*) expression was seen in liver tissue 24 hours after experimental infection with two different serotypes of *Actinobacillus pleuropneumoniae* in separate experiments (serotype 2 and serotype 6), compared to a shared control group of six animals (Fig. 5A). The down-regulation of the pig AGP gene amounted to 2–4 fold. Also looking at the liver response, experimental *Staphylococcus aureus* infection resulted in an approximate 4 fold reduction in *ORM1* expression at 30–48 hours after challenge, concomitantly with a pigMAP gene induction of around 20 fold and a haptoglobin gene expression fold change around two (Fig. 5B).

## Discussion

Purification of pig AGP, as followed by its cross-reactivity with polyclonal rabbit anti human AGP was relatively straightforward, taking advantage of the high solubility and the acidic nature of the protein, as compared to most other serum proteins. The purified protein gave rise to a broad band in SDS-PAGE. Molecular heterogeneity was also indicated in the first ion exchange chromatography step (cation exchange, CM Sepharose), as pig AGP was found both in the run-through from the CM Sepharose column and in eluted fractions obtained by increasing the pH of the buffer, corresponding to more acidic and less acidic isoforms, respectively. The more acidic isoforms had a slightly higher molecular weight than the less acidic isoforms (not shown). The mean molecular weight of the whole population was around 43 kDa showing up as one broad band in SDS-PAGE and as two parallel isoform series by 2-D electrophoresis (molecular weight: 44–55 kDa, see below). This suggests that part of the molecular heterogeneity of pig AGP may be explained by differences in the extent of sialic acid content, correlating increasing molecular weight with lower pI. There were additional sources of heterogeneity, however, as sialidase mediated removal of sialic acid did not noticeably sharpen the band in SDS PAGE. In contrast, complete N-deglycosylation led to a narrow band with no apparent molecular weight heterogeneity. Sialidase treatment resulted in an approximately 7 kDa decrease in molecular weight corresponding to around 22 sialic acid (*N*-acetyl neuraminic acid) moieties, suggesting that all five potential N-glycosylation sites were glycosylated with highly branched, complex-type N-glycans. This was confirmed by PNGase F treatment, reducing the molecular weight of pig AGP to 26 kDa, 17 kDa less than the intact protein. Assuming a typical tetra-antennary N-glycan molecular weight of 3606 Da, this corresponds to almost full occupancy of all five potential N-glycosylation sites with tetra-antennary glycans. The aglycan apparent molecular weight of pig AGP of 26 kDa is higher than the theoretical molecular weight as predicted from the sequence of the gene (21140 D) (Q29014, UniProt). This could be due to the presence of other post-translational modifications, including O-glycosylation or unusual glycan structures, as described in bovine and caprine AGP [50] possibly less amenable to hydrolysis by PNGase F. The isoelectric point (3.6–4.3) is lower than that predicted from the polypeptide alone (5.83), most likely due to the presence of sialic acid in the intact protein. In addition, phosphate groups may add to the low isoelectric point, as the polypeptide sequence contains several potential phosphorylation sites and phosphorylation was demonstrated for bovine AGP in a recent paper [51]. The presence of approximately 40% carbohydrate, the high sialic acid content and low pI are similar to AGPs of a range of other species [1], [2], [49], [52], [53]. Two-dimensional electrophoresis revealed the presence of distinct pI/molecular weight isoforms distributed on two dominant molecular weight subpopulations (44–55 kDa) and 5–7 different pI forms (3.6–4.3). This agrees well with a molecular model in which the molecular weight heterogeneity is accounted for by the polypeptide, while most of the rest of the heterogeneity stems from sialic acid (explaining the downward “slope” when going from higher to lower molecular weight and lower to higher pI) with some pI heterogeneity still residing in the polypeptide chain (charged/non-charged amino acids with little molecular weight difference) [54]. The same overall pI and molecular weight range and pattern of heterogenity was found by [7] and by [12] for Piétrain pig lectin (concanavalin A) binding AGP, identified by mass spectrometry in BALF.

Although immunization with desialylated pig AGP yielded less reactive antibodies (not shown) the monoclonal antibody produced (MAb 1.62) did not show any difference in reactivity with asialo- or deglycosylated pig AGP in Western blotting and reacted uniformly with all pI and molecular weight isoforms in 2-D electrophoresis blots. Thus, differences in concentrations were reflected reliably by the reactivity with MAb 1.62 on the blot. This was seen with semi-purified and purified forms as well as with a range of different sera. Likewise, the competitive ELISA based on MAb 1.62 was performing accurately with a range of sera as well as purified forms of pig AGP, including a commercial pig AGP standard, as seen by parallel titration curves (linearity under dilution). This is not trivial, as the differently glycosylated isoforms of pig AGP and thereby pig AGP populations of different compositions might theoretically have different reactivity with a given monoclonal antibody.

To our knowledge this is the first described monoclonal antibody based quantitative immunoassay for pig AGP, supplementing a commercially available single radial immunodiffusion assay based on polyclonal antibodies (Seikin Kagaku Institute Ltd.) and more complicated and semi-quantitative methods as e.g. densitometry of silver-stained 2-D gels [12].

Using this ELISA we show that perinatal AGP serum concentrations were elevated compared to the concentration in 1 month old pigs (Landrace/Yorkshire/Duroc/Hampshire crosses), confirming earlier reports in which a protein, tentatively identified as pig AGP and named fetospecific-like α-glycoprotein was found to be the major serum protein, accounting for 50% of the serum proteins at 12.7 mg/ml at birth and 0.24 mg/ml in adult (6 months) sera [8]. In a follow-up to this work pig AGP was identified and quantified by crossed immunoelectrophoresis using a polyclonal polyspecific rabbit anti pig serum and by analysis of amino acid and carbohydrate composition of the purified protein [9], confirming the preliminary characterization of pig AGP by [11]. Itoh et al. [23] determined pig AGP concentrations to be around 14 mg/ml in 1 day old Landrace pigs, dropping to around 0.7 mg/ml at 4 weeks of age. Developmental regulation was also demonstrated by [5] showing approximate 100 times decrease in the pig AGP mRNA level of fetal liver as compared to adult pig liver. We find 2–5 days old pigs to have a serum concentration of 6.6 mg/ml, possibly reflecting that a rapid decrease in pig AGP serum concentration occurred in the first few days after birth, and we determined the 1 month serum concentration to be 1.1 mg/ml. This is a typical adult level of pig AGP as found in a number of production breeds (Landrace, Duroc and Yorkshire) under standard production herd conditions, where concentration means range from 1.3 to 2.5 mg/ml. In contrast, concentrations in two breeds of minipigs (Ossabaw and Göttingen) were lower (0.3 mg/ml and 0.5 mg/ml, respectively) and a low concentration (0.6 mg/ml) was also found in a group of adult (9 months old) Yorkshire/Landrace pigs. Itoh et al. [23] reported an upper normal limit of 0.5 mg/ml for adult pigs (Landrace), Asai et al. [29] described concentrations to be in the range of 0.5–1 mg/ml in 4 weeks old SPF pigs, and Eckersall et al. [28] stated 0.3 to 0.6 mg/ml as the pig AGP concentration in 10 kg pigs. All of these studies used a single radial immunodiffusion kit from Saikin Kagaku Ltd. Lampreave and Pineiro [8] using the same method with an in-house standard measured 0.24 mg/ml as the mean concentration in normal pigs.

Pig AGP was found here to behave as a negative acute phase protein reacting with a 30–50% decrease in serum concentration during experimental *Streptococcus suis*, *Actinobacillus pleuropneumoniae*, and *Staphylococcus aureus* infections and during experimental, aseptic inflammation. At the same time the positive acute phase protein haptoglobin, determined in parallel in the same samples reached its peak concentration. This is noteworthy considering the diversity of the experimental treatments: *Streptococcus suis* is a Gram positive pathogen, administered subcutaneously and leading to arthritis [37], *Staphylococcus aureus* is a Gram positive pathogen, administered intravenously leading to severe sepsis with pronounced pulmonary lesions [38], *Actinobacillus pleuropneumoniae* is a Gram negative agent, administered intranasally [30[or through inhalation of an aerosol [34] and infecting the lung, and, finally, inflammation was created by localized subcutaneous aseptic injection of turpentine [35]. Thus, the negative acute phase response of pig AGP is not confined to a single type of infection or inflammation. Instead, it is indicated to be a general pig AGP acute phase response type resembling e.g. the response of apolipoprotein A-1 which is also a negative acute phase protein in the pig (and many other species) [55]. As AGP is generally described as a positive acute phase protein in all species, pig AGP has been used in a number of cases for monitoring acute phase responses (e.g. [23], [24], [25], [26]). However, Lampreave et al [27] and Eckersall et al. [28] both described AGP as not changing its serum concentration during the acute phase protein response to inflammation, and this was also reported by Asai et al. [29] after experimental porcine reproductive and respiratory syndrome virus infection. Kahlisch et al. [12] described pig AGP in BALF as a negatively reacting acute phase protein after experimental *Actinobacillus pleuropneumoniae* serotype 7 infection. However, this analysis was done on the subset of bronchoalveolar fluid glycoproteins binding to the lectin concanavalin A, and the negative response was only seen for the Piétrain breed as opposed to German Landrace (for which an increase was seen) and Hampshire breed (for which there was no change). The decrease in the Piétrain breed was concluded by the authors to probably not reflect the acute phase response of unfractionated serum AGP and was not further discussed. The source of BALF pig AGP was not investigated (infiltrating neutrophils as opposed to lung tissue derived) and quantification was done by densitometry of silver-stained 2-D gels. Finally, Yang et al. [31] reported the identification of a single down-regulated spot in two-dimensional electrophoresis of serum from pigs challenged with a highly pathogenic porcine respiratory and reproductive syndrome virus type as pig AGP, however these authors were not able to confirm down-regulation of overall pig AGP by one-dimensional Western blotting using an antibody raised against recombinantly expressed pig AGP [31].

We previously reported a moderate but significant down-regulation of the pig AGP gene (ORM1) in liver tissue at 24 hours after experimental infection with *Actinobacillus pleuropneumoniae* serotype 5b [21], and this is now confirmed to be reflected in the circulating concentrations of pig AGP protein. Furthermore we demonstrate here that hepatic expression of ORM1 was also decreased at 24 hours after experimental *Actinobacillus pleuropneumoniae* serotype 2 and 6 infection, dropping to 25% (serotype 6) and 50% (serotype 2) simultaneously with an increase in expression of the positive acute phase protein pig MAP of 10–20 times. Finally, the same degree of hepatic down-regulation of the pig ORM1 gene was shown after experimental *Staphylococcus aureus* infection, accompanied by a more than 10 fold increase in pig MAP expression.

Although these results clearly show that pig AGP is down-regulated as a part of the general acute phase response, other factors may be at play controlling this response. For example it was observed that experimental infection (aerosol) with pig influenza type A subtype H1N2 (Large White/German Landrace crossbreds, described in [47]) led to a small, positive pig AGP serum response (unpublished). Also, two other experimental infection studies (*Lawsonia intracellularis* (oral) in Danish Landrace/Yorkshire/Duroc crossbreds and *Toxoplasma gondii* (oral) in Danish Landrace/Yorkshire crosses) resulted in a well-differentiated acute phase protein response [56], [57], however caused no change in pig AGP serum concentrations as determined with the ELISA developed here (unpublished). It is also worth noting that obese Ossabaw minipigs (in contrast to obese Göttingen minipigs and obese domestic Landrace/Yorkshire crosses) showed an increase in pig AGP serum concentrations in obesity [58] resembling observations in mice and humans. It is not known how pig AGP reacts during the acute phase response in Ossabaw pigs.

The bronchoalveolar fluid glycoprotein results of [12] may indicate that there are differences between pig breeds in the acute phase response of pig AGP, and some, but not all, of the above observations may be explained by breed-dependent responses, however we have no systematic data to back this up. It is not unseen that different breeds of the same species may show different acute phase response patterns with specific acute phase proteins; this was for example observed for serum amyloid P protein in different mouse strains [59]. Also, the fact that pig AGP is dramatically regulated during development as shown by us and others [5], [8], [9] indicates that pig AGP synthesis is controlled by several different mechanisms. Finally, as reported in this paper, there seem to be big differences between non-induced serum pig AGP concentrations within and between a number of different pig breeds, as well as (especially) between differently reared pigs. It was especially intriguing that Yorkshire as well as Landrace pigs in normal herds had a much higher median pig AGP serum concentration than Landrace/Yorkshire crosses kept at experimental facilities. These data clearly warrant further, systematic studies on pig AGP during the acute phase response to specific infections comparing different pig breeds and different herds/breeders.

To summarize, we purified and extensively characterized purified pig AGP from pooled Landrace pig serum showing that, as in other species, it is an extensively glycosylated, very acidic microheterogeneous glycoprotein. Furthermore an immunoassay was established for the quantification of pig AGP and it was shown that this protein shows a hitherto undescribed acute phase response pattern during a range of different infections as well as after induced inflammation. This is the first description of AGP in any species as a true negative acute phase protein. Pig AGP serum concentrations, however, also seemed to be influenced by other factors, including possibly breed type, age and housing as well as handling. Its different types of responses, including the response to obesity in the Ossabaw breed [58] should be investigated further to establish if pig AGP might be useful as an acute phase biomarker with increased differential diagnostic abilities as compared to classically behaving acute phase proteins. Finally, the differences in the acute phase response of AGP (and other acute phase proteins) between species adds to the speculations on its biological function as well as on the biological function of the acute phase protein response itself.
